# The Influence of the Dispersion and Covalent Functionalization of CNTs on Electrical Conduction Under an Electric Field in LDPE/CNT Composites

**DOI:** 10.3390/polym17141940

**Published:** 2025-07-15

**Authors:** Xiaoli Wu, Ting Yin, Yi Yang, Wenyan Liu, Danping Wang, Libo Wan, Yijun Liao

**Affiliations:** 1 School of Materials and Environmental Engineering, Chengdu Technological University, Chengdu 611730, China; wxli1@cdtu.edu.cn (X.W.);; 2 School of Humanities and Design, Chengdu Technological University, Chengdu 611730, China; wanlibo@cdtu.edu.cn

**Keywords:** dispersion, covalent functionalization, CNT-based composites, electrical conduction, temperature, field emission

## Abstract

In this study, we comprehensively compare electrical conduction behavior under an applied electric field and electrical conductivity variation with temperature in low-density polyethylene (LDPE)/CNT composites with different dispersions and covalent functionalizations. Composites with different dispersions were prepared using solution and melt mixing processes. The solution-mixed composites exhibited better dispersion and higher electrical conductivity compared to the melt-mixed composites. At a high critical content (beyond the percolation threshold), the current–voltage (I–V) curve of the solution-mixed composites exhibited linear conduction behavior due to the formation of a continuous conductive network. In contrast, the melt-mixed composites exhibited nonlinear conduction behavior, with the conductive mechanism attributed to the field emission effect caused by poor interfacial contact between the CNTs. Additionally, LDPE/CNT-carboxyl (LDPE/CNT-COOH) and LDPE/CNT-hydroxy (LDPE/CNT-OH) composites demonstrated better dispersion but displayed lower electrical conductivity and similar nonlinear conduction behavior when compared to unmodified ones. This is attributed to the surface defects caused by the modification process, which lead to an increased energy barrier and a decreased transition frequency in the field emission effect. Furthermore, the temperature-dependent electrical conductivity results indicate that the variation trend in current with temperature differed among LDPE/CNT composites with different dispersions and covalent functionalizations. These differences were mainly influenced by the gap width between CNTs (mainly affected by dispersion and aspect ratio of CNTs), as well as the electrical conductivity of CNTs (mainly influenced by surface modification and intrinsic electrical conductivity of CNTs).

## 1. Introduction

Carbon nanotubes (CNTs) are one-dimensional nanotubular materials with a structure in which carbon atoms are predominantly *sp*^2^-hybridized [1,2]. In recent years, carbon nanotubes have attracted much attention due to their large aspect ratio, excellent electrical conductivity, and outstanding mechanical properties [3]. They are often incorporated as conductive particles into polymers to prepare conductive polymer composites, which are widely used in the fields of electromagnetic shielding [4,5], electric heating [6], antistaticity [7,8], etc. Due to the high aspect ratio and pronounced entanglement characteristics of CNTs, CNTs can easily agglomerate within a matrix. Thus, the dispersion of CNTs is a critical factor that significantly influences the electrical conductivity and mechanical properties of CNT–polymer composites [9,10].

The dispersion of conductive fillers in polymer composites is influenced by various factors, including the preparation method (including shear force) [11,12], surface modification, the length of CNTs [13], the viscosity of polymers [14], etc. For instance, Bai et al. [13] investigated the effect of nanotube length and aggregate size on the mechanical and electrical properties of composites and reported that the length and aggregate size had a very clear impact on the dispersion and dielectric properties of the composites. Sui et al. [15] found that the effect of preparation methods on dispersion and electrical conductivity is related to the content of CNTs. Faraguna et al. [16] prepared differently functionalized-CNT–polystyrene (PS) nanocomposites by melt and solution mixing, revealing that the solution-mixed nanocomposites exhibited lower percolation thresholds due to better dispersion compared to the melt-mixed composites. These studies have provided important guidance for the effect of CNT dispersion on the electrical conductivity and percolation threshold of CNT–polymer composites. However, the investigation of the influence of CNT dispersion on electrical conduction behavior under electric fields remains sluggish.

Most previous studies reported that the electrical conductivity of polymer composites increased with the increase in electric field when the filler content approached the percolation threshold [17,18,19]. That is, the intensity current–voltage (I–V) curve shows a nonlinear characteristic [20]. Previous studies have suggested that the nonlinearity of each element may be responsible for its intrinsically nonlinear characteristic [21]. Alternatively, it has been proposed that the transition from insulating to conducting channels under strong local fields could account for nonlinear electrical conductivity [22]. This transition is likely due to the nonlinear hopping or tunneling of charge carriers. He et al. [23] have demonstrated that a combination of nonlinear conduction induced by internal field emission and linear conduction contributed by a conducting network governs the overall electrical conduction process in percolating systems. Although these studies have described nonlinear electrical conduction for composites with different contents near the percolation threshold, none of them have focused on the investigation of electrical conduction under an electric field for composites with different dispersion states.

In most cases, it is necessary to chemically modify the surface of CNTs in order to achieve good dispersion in CNT–polymer composites and strong interfacial adhesion between CNTs and polymer matrices [24,25,26,27]. However, chemical modification processes may destroy the surface structure of CNTs. For example, Sui et al. [15] reported that carboxyl–CNT (COOH-CNT) had better dispersion than unmodified CNT, while COOH-CNT/thermoplastic polyether urethanes (TPU) composites had lower conductivity and higher percolation thresholds, which can be attributed to the structure defects and the reduced length of CNTs caused by the acidification process. Similarly, Trinh et al. [28] also demonstrated that COOH-CNT nanofluids showed lower electrical conductivity due to structure defects in CNTs, in comparison to unmodified CNT nanofluids, which even revealed a higher agglomerated size. These investigations have focused on the effect of surface chemical modification on dispersion, mechanical properties, and thermal and electrical conductivity [26,27,29]. However, the influence of surface chemical modification on electrical conduction behavior under electric fields is not very clear. Furthermore, there is a lack of research exploring how the covalent functionalization method affects the electrical conductivity variation as a function temperature for CNT based composites.

In order to systematically investigate the effect of CNT dispersion on electrical conduction behavior under the applied electric field and the temperature-dependent electrical conductivity variation, the low-density polyethylene (LDPE)/CNT composites with different dispersion states were prepared by the melt and solution mixing methods. The reason for using LDPE as the matrix is due to its ability to melt at high temperatures and dissolve in the solvent xylene [30], and its excellent mechanical properties [31]. What is more, COOH-CNT and OH-CNT were employed as the conductive fillers of the composites to systematically investigate the influence of surface chemical modification on CNT dispersion, electrical conduction behavior under an applied electric field, and temperature-dependent electrical conductivity variation.

## 2. Experimental Section

### 2.1. Materials

Low-density polyethylene (LDPE) was provided by Dongguan Shanhe Plastic Technology Co., Ltd., Dongguan, China. Multi-walled CNT (TNM1), the corresponding carboxyl-CNT (COOH-CNT, TNMC5) and hydroxy-CNT (OH-CNT, TNMH5) with 5–15 nm diameter and 98% purity were provided by the Chengdu Organic Chemicals Pty Ltd., Chengdu, China. Xylene (AR grade) was purchased from Chengdu Jinshan Chemical Co., LTD., Chengdu, China. Anhydrous ethanol (AR grade) was provided by Chengdu Colon Chemical Co., LTD., Chengdu, China.

### 2.2. Preparation of Composites

For the melt mixing process, the dried unmodified CNT and LDPE were mixed in a Torque Rheometer (Rheoccord System 40, Haake Technik GmbH, Vreden, Germany) at 200 °C. The mixing speed was set at 60 and 120 rpm, respectively. Then the LDPE/CNT composites were hot-pressed at 190 °C and 10 MPa for 5 min into a sheet with a size of 50 × 50 × 2 mm^3^ for different characterizations. A series of LDPE/CNT composites with different volume fractions of unmodified CNT (0.7, 1.4, 2, 2.8, 3.5, 4.5, 5, and 6 vol.%) were prepared. The LDPE/CNT-COOH and LDPE/CNT-OH composites with the same volume fraction were fabricated using the above process with a mixing speed of 120 rpm.

For the solution mixing process, a certain amount of CNTs was suspended in ethanol by sonication. The LDPE was dissolved in the xylene at 130 °C by magnetic stirring. When the mixture became transparent, the CNT/ethanol suspension was slowly dropped into the LDPE/xylene solution, and the mixture was stirred for 1 h. Subsequently, the mixture was filtered and vacuum dried at 60 °C for 72 h to remove the xylene. By measuring the weight, we confirmed that the xylene had been completely evaporated. Finally, the composite was put into the mold and hot pressed at 190 °C and 10 MPa for 20 min to obtain a plate-like sample with a size of 50 × 50 × 2 mm^3^. The volume fraction of CNT in the LDPE/CNT composites prepared by the solution mixing method is 0.7, 1.4, 2, 2.8, 3.5, 4.5, 5, and 6 vol.%.

The obtained LDPE/CNT composites prepared by different methods were marked as CNT-60-x, CNT-120-x, and CNT-solution-x, respectively, where x stands for the CNT content. The composites with and without grafting groups were named as CNT-x, CNT-COOH-x, and CNT-OH-x, respectively, where x represented the CNT content.

### 2.3. Material Characterization

The cross-sectional morphology of the LDPE/CNT composites was characterized by scanning electron microscopy (SEM, Helios 5 CX, Thermo Scientific, Waltham, MA, USA). The cross-section was obtained by brittle fracture in liquid nitrogen. The tensile and flexural tests were conducted using an electronic universal testing machine (68TM-10, Instron Corporation, Boston, MA, USA). The specimens have a size of 50 × 10 × 2 mm^3^. The loading rate was 50 mm/min and the gauge length was 25 mm. The flexural test was carried out with a loading rate of 10 mm/min, and the flexural displacement was set to 8 mm, at which point the test stopped.

### 2.4. Electrical Performance Characterizations

The volume electrical conductivity of the composites was examined using the RTS-11 four-probe resistivity tester (Shenzhen Junda Times Instrument, Shenzhen, China) when the electrical conductivity was above 10^−6^ S/cm and the ZST121 electric resistance meter (Avic Xi’an Aircraft Industry Group Company Ltd., Xi’an, China) when the electrical conductivity was below 10^−6^ S/cm. The electrical conductivity of CNTs was examined by the RTS-11 four-probe resistivity tester after the CNTs were pressed into a sheet at 15 MPa. The electrical conductivity of the sample was calculated using the following equation:

(1) $$\sigma =\frac{t}{R\times S}$$

where σ is the electrical conductivity, t is the thickness, R is the electrical resistance, and S is the area of the samples.

The electrical conduction was carried out using a DC voltage source (DC1203D, Dingce Power Technology, Dongguan, China). The electrical conduction and the volume electrical resistivity at different temperatures (−40–80 °C) were measured in a freezer and a drying oven. The connection area of the equipment was wrapped with insulation foam to ensure the accuracy of the temperature.

The effective aspect ratio of CNTs in the matrix was calculated by substituting the percolation threshold into the following equations, which are given below [32,33,34]:

(2) $$\psi_{c}=\frac{18{S^{2}}_{11}-9S_{11}}{18{S^{2}}_{11}-3S_{11}-4}$$

(3) $$S_{11}=S_{22}=\left\{\begin{array}{l} \frac{\alpha }{2{\left(1-\alpha^{2}\right)}^{\frac{3}{2}}}\left[\cos^{-1}\alpha -\alpha {\left(1-\alpha^{2}\right)}^{\frac{1}{2}}\right],\alpha <1\\ \frac{\alpha }{2{\left(\alpha^{2}-1\right)}^{\frac{3}{2}}}\left[\alpha {\left(1-\alpha^{2}\right)}^{\frac{1}{2}}-co{sh}^{-1}\alpha \right],\alpha >1 \end{array}\right.$$

where *ψ_c_* is the volume fraction of the percolation threshold, *α* is the aspect ratio of CNT, and *S*_11_ and *S*_22_ are the components of the shape-dependent depolarization tensor *S* of associated phases. For fillers with spherical symmetric shape, *S*_11_ = *S*_22_ = 1/3 [32]. For fillers with an asymmetric axis 3, such as carbon nanotubes, graphene, and other ellipsoidal fillers, *S*_11_ can be calculated by the formula mentioned above [33]. Previous research reports that the percolation threshold (*ψ_c_*) is a strictly geometrical parameter [34], which represents the onset of the connective networks through the composite. It is only related to the aspect ratio of the fillers and can be used to calculate *ψ_c_* [35,36]. Therefore, in this study, we inversely used the above equations to calculate the effective aspect ratio of CNTs in the matrix.

## 3. Results and Discussion

### 3.1. The Effect of CNT Dispersion on the Electrical Conduction Under an Electric Field for the LDPE/CNT Composites

**Figure 1:**
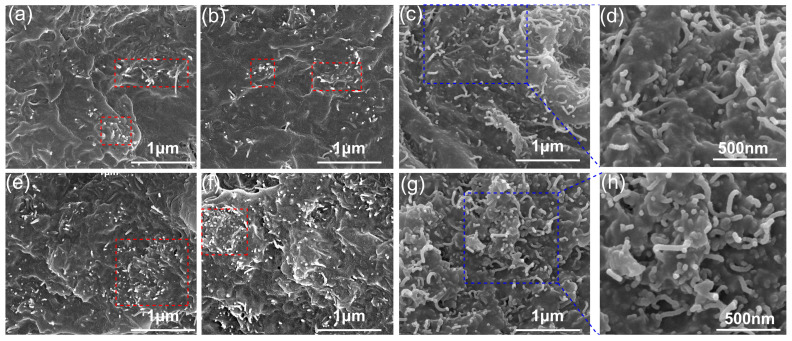
The SEM morphology of the LDPE/CNT composites prepared by different mixing processes: (**a**) CNT-60-3.5 100,000×; (**b**) CNT-120-3.5 100,000×; (**c**) CNT-Solution-3.5 100,000×; (**d**) CNT-Solution-3.5 200,000×; (**e**) CNT-60-6 100,000×; (**f**) CNT-120-6 100,000×; (**g**) CNT-Solution-6 100,000×; (**h**) CNT-Solution-6 200,000×.

The LDPE/CNT composites with different dispersion states were fabricated using melt and solution mixing methods. The dispersion of the LDPE/CNT composites was observed by SEM. At a low volume fraction (3.5 vol.%), for the melt-mixed composites, as depicted in Figure 1a, the distribution of CNTs was less uniform, with some agglomerations observed for the composites subjected to a stirring speed of 60 rpm (see the red dashed boxes). When the stirring speed increased to 120 rpm, the amount of the CNT agglomerates reduced, as depicted in Figure 1b, exhibiting a better CNT dispersion primarily due to enhanced shear force. The solution-mixed composites exhibit a more uniform dispersion, without any noticeable agglomerates (Figure 1c,d). As shown in Figure 1c,d, the CNTs were observed to be in contact with each other, thereby establishing a continuous conductive network within the solution-mixed composites. When the volume fraction was increased to 6 vol.% (Figure 1e–h), the agglomeration of CNTs in the melt-mixed composites was more obvious, while the solution-mixed composites still exhibited the best dispersion. For the melt-mixed composites with a stirring speed of 120 rpm, the CNTs formed interconnected conduction paths but failed to establish a continuous network. When the stirring speed decreased to 60 rpm, obviously isolated CNTs were observed (Figure 1e). The aforementioned results demonstrated that the dispersion of CNT in the solution-mixed composite surpassed that in the melt-mixed composite, and that an increase in shear force can enhance CNT dispersion. This result was in agreement with other previous studies [11,16,37]. They have reported that a better dispersion enabled CNTs to form continuous conducting networks more easily.

**Figure 2:**
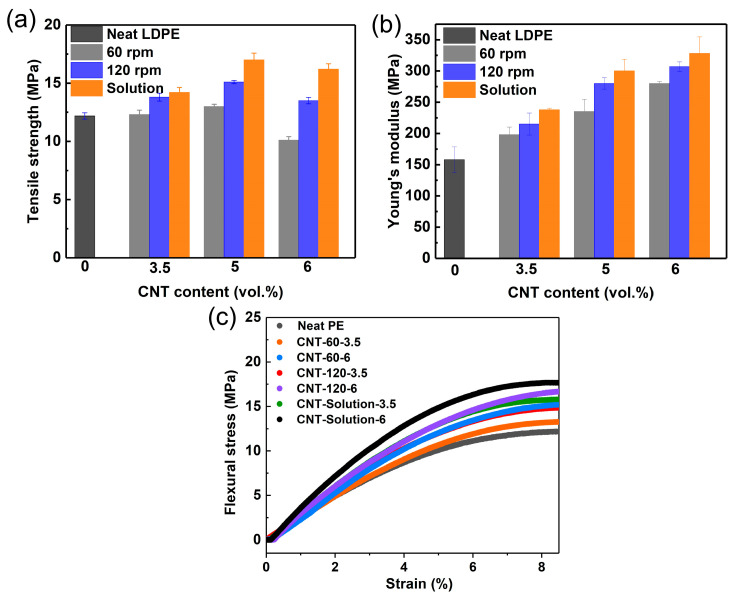
The mechanical strength of the LDPE/CNT composites prepared by different mixing processes at different CNT volume fractions: (**a**) tensile modulus; (**b**) the ultimate tensile strength; (**c**) the flexural strength and strain curves.

In order to further confirm the dispersion state of the LDPE/CNT composites with different preparation methods, the mechanical properties were tested. Figure 2a,b show the tensile modulus and tensile strength of the LDPE/CNT composites with different preparation methods, respectively. It can be clearly observed that the solution-mixed LDPE/CNT composites exhibit the highest tensile modulus and strength at both low and high contents. The melt-mixed composites with a stirring speed of 120 rpm exhibited higher tensile modulus and strength compared to those with a stirring speed of 60 rpm. These results should be related to the homogeneity of CNT. The excellent dispersion of CNT in LDPE resulted in the interconnection between the polymer matrix and CNTs, thereby reducing the slip and enhancing the mechanical strength of the composites [38,39]. In addition, a decline in tensile strength was observed at 6 vol.% CNTs, which was likely attributed to the more agglomeration resulting in poor stress transfer [15]. Figure 2c shows the flexural strength of the LDPE/CNT composites with different preparation methods. The solution-mixed LDPE/CNT composites also exhibited the highest flexural strength at both low and high contents. These results suggest that the solution-mixed LDPE/CNT composite exhibited superior dispersion, while the melt-mixed composite with a stirring speed of 60 rpm showed the poorest dispersion.

**Figure 3:**
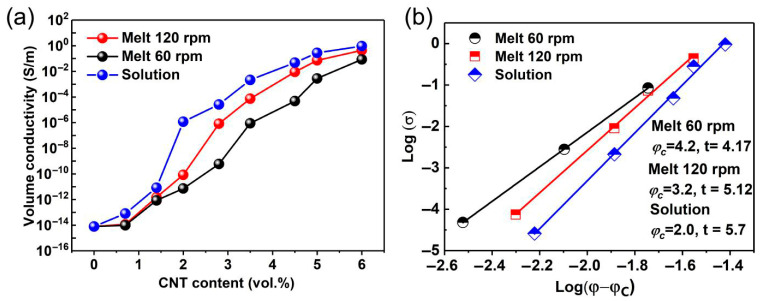
(**a**) The volume conductivity (*σ*) of the LDPE/CNT composites prepared by different mixing processes; (**b**) the Log plots of *σ* versus *ψ-ψ_c_* and their fit curves.

Figure 3a shows the volume electrical conductivity of the LDPE/CNT composites prepared by different methods. For the melt-mixed composites, the electrical conductivity of the composite with a stirring speed of 120 rpm was higher than that of the composite with a stirring speed of 60 rpm. The solution-mixed composites demonstrated a higher electrical conductivity than the melt-mixed composites. The percolation threshold was calculated using the classical percolation formula, as delineated below [40]:

Σ = σ_f_ (*ψ* − *ψ_c_*)^t^ (*ψ > ψ_c_*) (4)

where *ψ* is the volume fraction of the filler, *ψ_c_* is the percolation threshold, and *σ* and *σ_f_* are the conductivities of the composite and filler, respectively. In addition, *t* is a critical power exponent that depends on the dimensionality of the conducting network and typically takes the value of ~1.33 for two-dimensional (2D) systems (e.g., in coatings) [41], For three-dimensional (3D) systems, this is usually taken as ~2 [42]. Log values were taken for each side of the equation, and a linear line fit was used. The fitting results are shown in Figure 3b. The percolation thresholds of the melt-mixed composites with stirring speeds of 60 and 120 rpm were fitted to be 4.2 and 3.2 vol.%, respectively. Moreover, the corresponding *t*-values were significantly high at 4.17 and 5.12. Combined with the SEM results, the reason for such a high t-value was likely attributed to the formation of tunneling percolation systems [37,43], which were formed by conductive particles located apart from each other and enclosed by the polymer matrix. This system may arise from extensive CNT aggregations in the melt-mixed composites. For the solution-mixed composites, the percolation threshold (2 vol.%) was obviously lower than that of the melt-mixed composites. Moreover, the t-value reached as high as 5.7, which was significantly higher than that of the melt-mixed composites. Such a high *t*-value was likely due to the formation of a 3D conductive network (see Figure 1h) as well as the presence of small CNT aggregations. These results further confirmed the differences in the dispersion state of the LDPE/CNT composites prepared by different methods.

**Figure 4:**
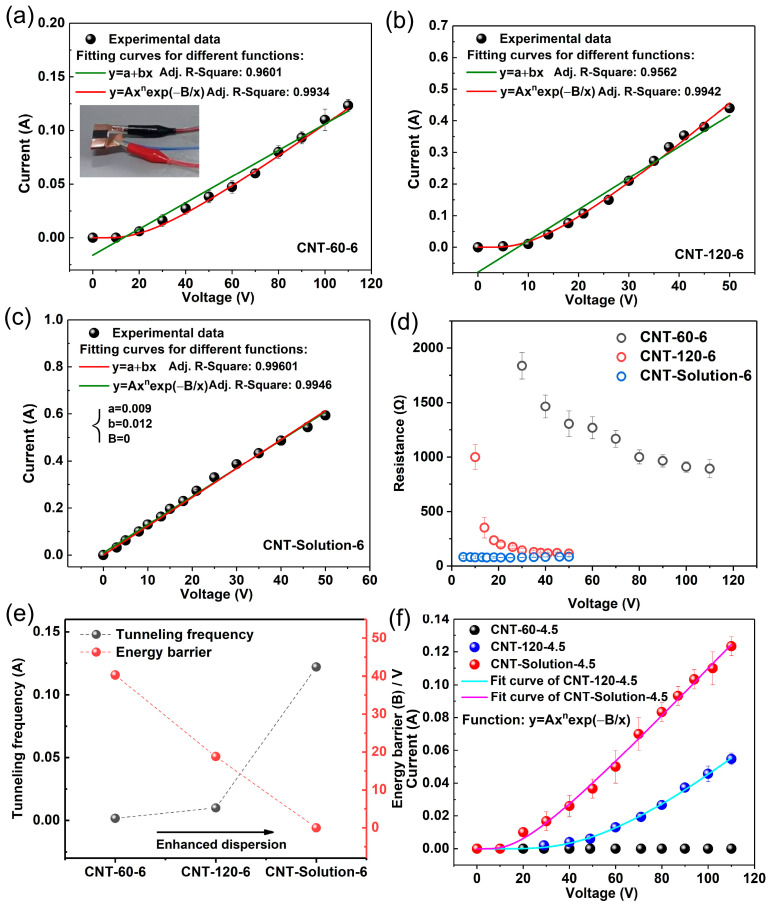
(**a**–**c**) The current intensity variation as a function of applied voltage for the LDPE/CNT composites prepared by different mixing processes: (**a**) CNT-60-6; (**b**) CNT-120-6; (**c**) CNT-Solution-6. The solid lines correspond to the best linear-fitting result and fitted curve from the electric field emission equation, respectively. (**d**) The resistance variation as a function of applied voltage; (**e**) the fitted parameters of transition probability A and the energy barrier B; (**f**) the current intensity variation as a function of applied voltage and fitted curve from the electric field emission equation for the LDPE/CNT composites prepared by different mixing process at a volume fraction of 4.5 vol.%.

The electrical conduction under an electric field of the LDPE/CNT composites with different preparation methods was examined at ambient temperature. The composites with a volume fraction of 6 vol.% were selected for testing due to their content exceeding the percolation threshold and showing conductive behavior (*σ* < 10^−4^ S/m) [44]. The current–voltage (I–V) curves are shown in Figure 4a–c. The solution-mixed composite demonstrated the highest current value at a given voltage among the three groups of composites, owing to its superior electrical conductivity. The current of all three groups of composites exhibited an increasing trend with the applied voltage; however, it is evident that the I–V curves of these three groups have obvious differences. For the solution-mixed composite, the I–V curve showed a linear characteristic, whereas the I–V curves of the melt-mixed composites exhibited a nonlinear variation. Moreover, the resistance (R) of the melt-mixed composites decreased with the applied voltage (Figure 4d). Previous studies have reported that the nonlinear variation in percolated composites primarily arose from electron tunneling induced by the field emission effect occurring between isolated fillers [22]. The internal field emission effect was proposed by Zener in the 1930s [45], through which a large number of isolated conductive particles that are not connected to the whole conductive network can also participate in the conduction process by jumping over the insulating barriers through electron tunneling. The curves were fitted using linear and field emission formulas, respectively. The specific formulas employed are as follows:

*I* = *a* + *bV* (5)

**Figure 5:**
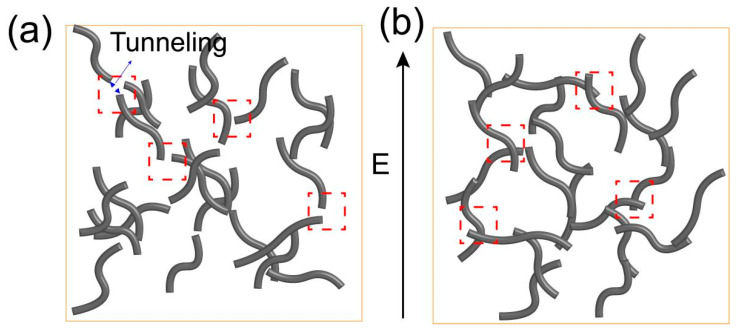
(**a**) The schematic diagram of the LDPE/CNT composites with CNT aggregates; (**b**) the schematic diagram of the LDPE/CNT composites with good dispersion.

*I* = *AV^n^exp (−B/V)* (6)

where *I* is the current intensity; *V* is the applied voltage; *a*, *b*, *A*, *B,* and *n* are constants; and *n* is usually between 1 and 3 depending on various corrections included in the theory, such as the effects of image fields or Coulombic forces [46]. *A* is a function of tunneling frequency, i.e., the number of attempts to cross the barrier per second, and *B* represents the energy barrier between the insulating matrix and the conducting fillers, which is mainly related to the gap width between the conducting fillers [47]. In this study, the fitted curves and parameters for the two formulas are also shown in Figure 4a–c. The I–V curves of the melt-mixed composites with stirring speeds of 60 and 120 rpm exhibited a closer adherence to the field emission equation as compared to that of the linear one. This result suggests that the dominant conductive mechanism of melt-mixed composites is the field emission effect. This can be attributed to non-uniform dispersion resulting in insufficient interconnection between CNTs, see schematic diagram in Figure 5a. Moreover, it is apparent that the composite stirred at 120 rpm exhibits a higher tunneling frequency (*A*) and lower potential barrier (*B*) compared to the one stirred at 60 rpm (Figure 4e). This can be attributed to the better dispersion, resulting in a narrower gap width between CNTs in the former.

The *I–V* curve of the solution-mixed composite demonstrated a linear characteristic, indicating that the conductivity mechanism follows the percolation theory. This finding was different from the melt-mixed composites. It was likely arising from the more effective interconnection between CNTs, resulting in a reduction in gap width between CNTs, see schematic diagram in Figure 5b. This results in a decrease in potential barrier (*B*) derived from the field emission formula, as shown in Table 1. The *B* value became zero, leading to the transformation of the field emission equation into linear equation of *I* = 0.012 *V*. When the CNT content decreased to 4.5 vol.%, the conductive mechanism of the solution-mixed composite became the field emission effect, as evidenced by the experimental data and its fitting curve depicted in Figure 4f. *A* higher *B* value was obtained (Table 1), which was likely due to the larger gap width between CNTs. Therefore, the above results indicate that the CNT dispersion appears to exhibit a similar effect as the filler content, influencing not only the electrical conductivity and percolation threshold but also the gap width of CNTs and the electrical conduction behavior under the electric field.

**Figure 6:**
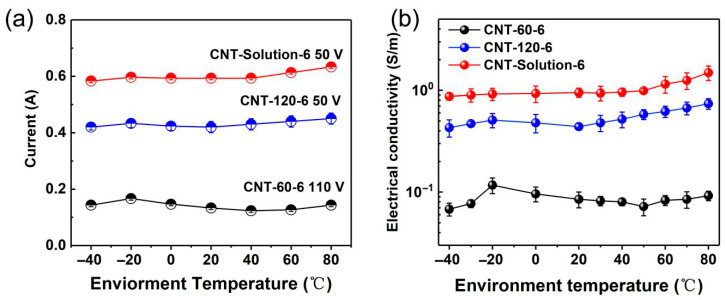
(**a**) The current variation as a function of temperature for the LDPE/CNT composites prepared by different mixing processes; (**b**) the electrical conductivity variation as a function of temperature for the LDPE/CNT composites prepared by different mixing processes.

In engineering applications, the variation in electrical conductivity as a function of temperature serves as a crucial indicator. Therefore, in this study, the electrical properties of the LDPE/CNT composites with different dispersion states at different environmental temperatures were investigated. Figure 6a shows the current variation in the composites with content of 6 vol.% prepared by different preparation methods at environment temperatures of −40–80 °C. It can be seen that there is an obvious difference in the current variation trends of the three LDPE/CNT composites at their respective maximum operating voltages. When the environmental temperature decreased from 20 to −20 °C, the current values of these composites exhibited an increasing trend. The melt-mixed composite with a stirring speed of 60 rpm showed a maximum increase value. It can be attributed to its poorer dispersion, resulting in inadequate contact between CNTs. Consequently, when the temperature was reduced, the gap width between CNTs was significantly decreased due to the matrix contracting to some extent as the temperature dropped [48]. As the environmental temperature decreased to −40 °C, the current values of all three groups of composites showed a decreasing trend, which was mainly related to the decrease in the energy of charge carriers at low temperatures [49].

As the temperature increased from 20 °C to 80 °C, the current value of the melt-mixed composite with a stirring speed of 60 rpm initially exhibited a decreasing trend, followed by a slight increase. The decrease may be attributed to the increase in the gap width between CNTs induced by the thermal expansion of the polymer matrix as the temperature rose [50]. The increase may be due to the fact that the carrier energy was elevated at higher temperatures [51,52]. The increasing trend also occurred in the current values of melt-mixed composites with a stirring speed of 120 rpm and the solution-mixed composites. It is interesting that these two groups did not exhibit a declining trend, potentially due to the better dispersion state (more effective contact) resulting in less variation in gap width between CNTs, see schematic diagram in Figure 5. The above results are further confirmed by the electrical conductivity variation with temperature, see Figure 6b. It can be concluded that the dispersion states of CNTs have a significant impact on electrical conduction and conductivity of the composites with temperature.

### 3.2. The Effect of Covalent Functionalization on the Dispersion of CNT and the Electrical Conduction Under an Electric Field for the LDPE/CNT Composites

**Figure 7:**
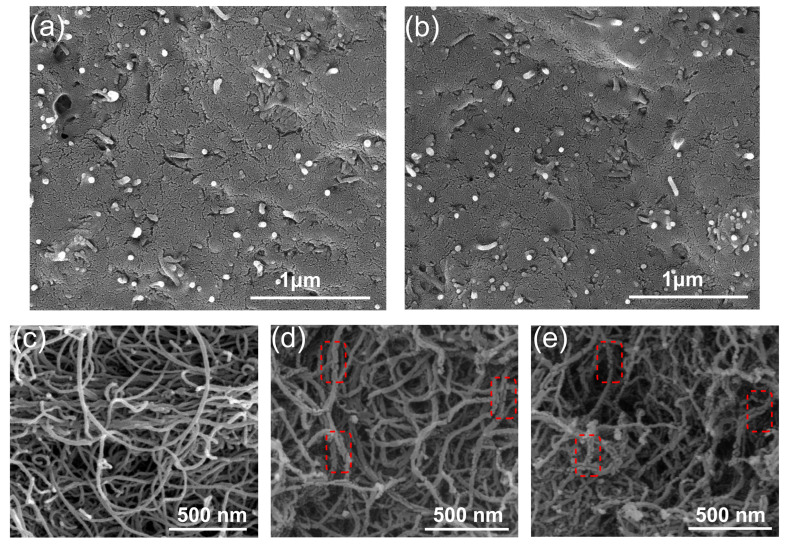
The SEM morphology of (**a**) LDPE/CNT-COOH-6 composite; (**b**) LDPE/CNT-OH-6 composite; (**c**) CNT; (**d**) COOH-CNT; (**e**) OH-CNT.

The SEM morphologies of the LDPE/CNT-COOH and LDPE/CNT-OH composites with a volume fraction of 6 vol.% are shown in Figure 7a,b. It can be seen that there is no obvious difference in the dispersion of the LDPE/CNT-COOH and LDPE/CNT-OH composites. When compared to the unmodified LDPE/CNT composites, they exhibited a smaller agglomeration size and more uniform distribution of CNTs within the matrix. However, it is worth noting that the LDPE/CNT-COOH and LDPE/CNT-OH composites have formed conductive paths, while there was a certain gap width between the conductive chains, and there were some isolated CNTs. In addition, the gap width between CNTs seemed to be larger than that of the unmodified LDPE/CNT composites. It could be due to the destruction of the surface structure of the CNT during the acidification modification and the reduction in length, which was determined by the SEM morphology of COOH-CNT and OH-CNT (Figure 7c,d).

**Figure 8:**
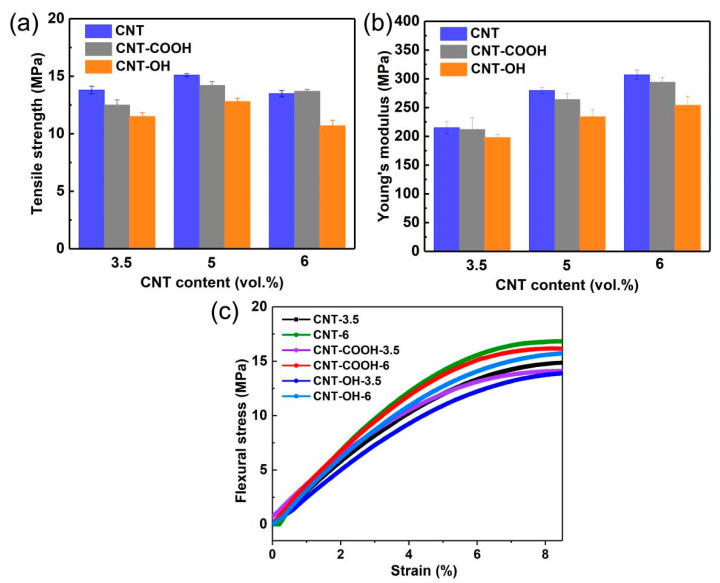
The mechanical strength of the modified CNT/ LDPE composites at different CNT volume fractions: (**a**) tensile modulus; (**b**) the ultimate tensile strength; (**c**) the flexural strength and strain curves.

Figure 8 shows the tensile modulus, tensile strength, and flexural strength–strain curves of the LDPE/CNT-COOH and LDPE/CNT-OH composites, respectively. The result of the unmodified LDPE/CNT composite is also displayed for comparison. It is clearly observed that the LDPE/CNT-COOH and LDPE/CNT-OH composites exhibit lower tensile modulus and tensile strength compared to those of unmodified composites at both low and high contents, see Figure 8a,b. Figure 8c shows the flexural strength of the LDPE/CNT-COOH and LDPE/CNT-OH composites. It can be seen that the flexural strengths of the LDPE/CNT-COOH and LDPE/CNT-OH composites are also lower than the unmodified composites at both low and high contents. The poor reinforcing effect of COOH-CNT and OH-CNT may be attributed to the reduction in CNT length and the many structural defects. This indicates that the uniform dispersion of CNTs does not appear to be a critical factor influencing the enhancement of mechanical properties in the composites as compared to the structural defects induced by the chemical modification process.

**Figure 9:**
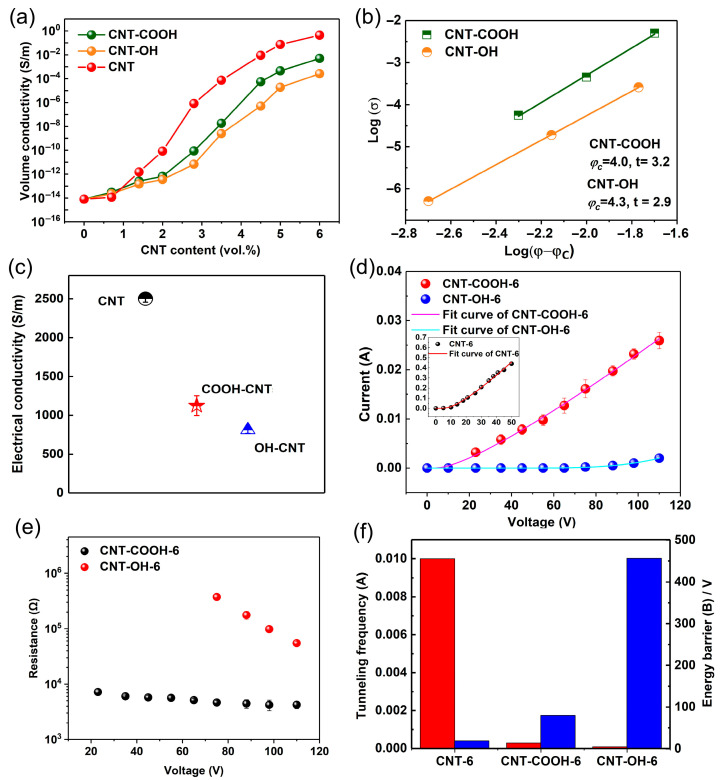
(**a**) The volume conductivity (*σ*) of the modified LDPE/CNT composites. (**b**) The Log plots of *σ* versus *ψ-ψ_c_* and their fit curves. (**c**) The volume conductivity of CNT, COOH-CNT, and OH-CNT pressed sheets. (**d**) The current intensity variation as a function of applied voltage for the modified LDPE/CNT composites and unmodified LDPE/CNT composites. The solid lines correspond to the best-fitted curve from the electric field emission equation. (**e**) The resistance variation as a function of applied voltage. (**f**) The fitted parameters of transition probability *A* and the energy barrier *B*.

Figure 9a,b show the volume electrical resistivity and the percolation threshold of LDPE/CNT-COOH and LDPE/CNT-OH composites. It can be seen that the LDPE/CNT-COOH and LDPE/CNT-OH composites exhibit lower electrical conductivity and higher percolation thresholds compared with the unmodified LDPE/CNT composite. The percolation thresholds of the LDPE/CNT-COOH and LDPE/CNT-OH composites were as high as 4.0 and 4.3 vol.%, respectively. This result could be attributed to two factors. Firstly, the presence of structural defects led to a decrease in electrical conductivity [15,29,53]. This was supported by the results of the electrical conductivity of the modified and unmodified carbon nanotubes (CNTs), as shown in Figure 9c. The data indicates that the electrical conductivity of COOH-CNT (1124.3 S/m) and OH-CNT (813.6 S/m) is significantly lower than that of pristine CNT (2501.7 S/m). Secondly, the existence of structural defects caused a reduction in length, which affected the formation of conductive networks [54]. This was supported by the calculated effective aspect ratios of carbon nanotubes (CNTs) in the matrix, obtained using Equations (2)–(4). These values were 25.0, 23.2, and 31.7 for LDPE/CNT-COOH, LDPE/CNT-OH, and unmodified LDPE/CNT composites, respectively.

The current–voltage (I–V) curves of the LDPE/CNT-COOH and LDPE/CNT-OH composites with a fraction volume of 6.0 vol.% are depicted in Figure 9d. It can be observed that the LDPE/CNT-COOH and LDPE/CNT-OH composites show lower current values than the unmodified LDPE/CNT composite. The I–V curves of the two modified LDPE/CNT composites exhibited nonlinear characteristics (Figure 9d,e) and were fitted to the field emission equation, which was similar to that of the LDPE/CNT composite. Differently, the two modified composites exhibit significantly larger values of potential barrier (*B*) and lower values of tunneling frequency (*A*) compared with the unmodified composite (Figure 9f). This could be due to their reduced charge carrier concentration and mobility of CNT caused by surface defects, leading to a decrease in tunneling frequency (*A*). Additionally, the reduction in CNT length increases the gap width between CNTs, leading to an increase in the potential barriers (*B*). These results indicate that the structural defects induced by the chemical modification process seem to be a more critical factor influencing the enhancement of electrical properties in the composites as compared to that of the uniform dispersion of CNTs.

**Figure 10:**
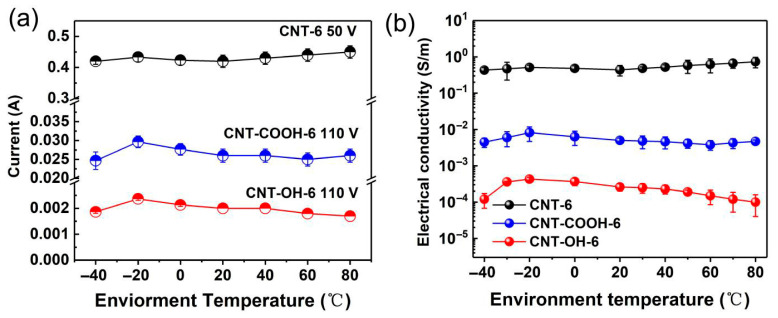
(**a**) The current variation as a function of temperature for the modified LDPE/CNT composites; (**b**) the electrical conductivity variation as a function of temperature for the modified LDPE/CNT composites.

**Figure 11:**
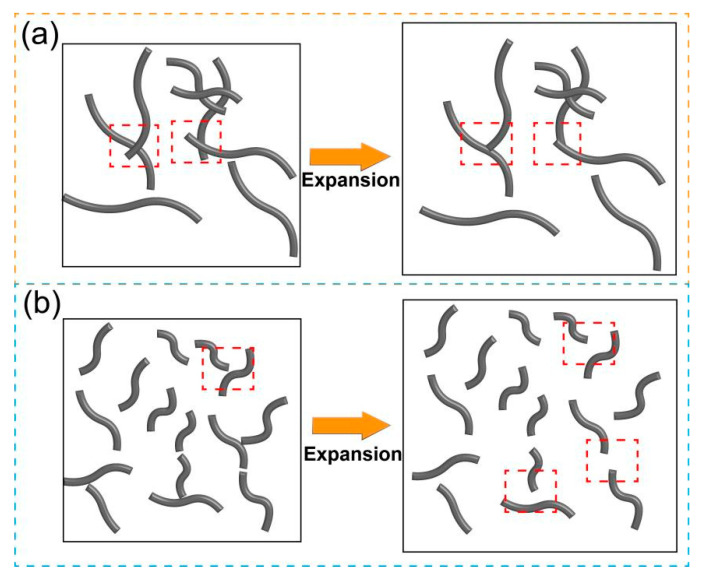
The schematic diagram pre- and post-expansion for (**a**) the unmodified LDPE/CNT composites with long CNT and several aggregates and (**b**) the modified LDPE/CNT composites with good dispersion but short CNT.

Figure 10a shows the current variation in the LDPE/CNT-COOH and LDPE/CNT-OH composites with a volume fraction of 6 vol.% at environmental temperatures of −40–80 °C. It can be seen that the two modified LDPE/CNT composites exhibit different current variations with environmental temperature at their respective operating voltages. When the environmental temperature changed from 20 °C to −40 °C, the current variations in the two modified LDPE/CNT composites initially increased and subsequently decreased, exhibiting a similar trend to that observed in the unmodified LDPE/CNT composite. It is worth noting that the change in slope of the two modified composites is higher, which is likely attributed to the reduced effective aspect ratio of CNT. The shorter CNTs resulted in a larger gap width between CNTs compared to long CNTs [55]. While the larger gap width between CNTs may lead to more significant changes during heating or cooling, as illustrated in the schematic diagram of Figure 11.

When the environmental temperatures increased from 20 °C to 60 °C, the current values of both the modified composites decreased with the increase in temperature, which was different from the results of the unmodified LDPE/CNT composite. It could be attributed to the expansion of the matrix at higher temperatures, leading to an increased gap width between CNTs. As the environmental temperature rose from 60 °C to 80 °C, the LDPE/CNT-COOH composite exhibited a slight increasing trend in current. This behavior was likely due to the effect of the increase in energy of the carriers caused by elevated temperature, surpassing the effect of the gap width variation. However, the LDPE/CNT-OH composite continued to show a decreasing trend in current, primarily due to its more severe structural disruption and lower electrical conductivity of CNTs. The electrical conductivity of both the modified composites exhibited similar variations with temperature (refer to Figure 10b). Consequently, we can conclude that the variations in electrical conductivity with temperature are mainly influenced by the gap width between CNTs (mainly affected by dispersion and aspect ratio of CNTs) as well as the electrical conductivity of CNTs (mainly influenced by surface modification and intrinsic electrical conductivity of CNTs).

## 4. Conclusions

In summary, we have studied the effect of CNT dispersion and covalent functionalization on the electrical conduction behavior under the applied electric field for LDPE/CNT composites for the first time. At a high critical content beyond the percolation threshold, the I–V curve showed a linear characteristic for the solution-mixed composite due to its continuous network of CNTs. While the melt-mixed composites exhibited a nonlinear characteristic, the conductive mechanism was found to be the field emission effect, which was likely contributed to the poor contact of the CNTs. On the other hand, the LDPE/CNT-COOH and LDPE/CNT-OH composites demonstrated better dispersion, but lower electrical conductivity and similar nonlinear conduction behavior compared to that of the unmodified ones. This was attributed to the surface defects caused by the covalent functionalization process, which led to an increased energy barrier and a reduced transition frequency in the field emission effect. Furthermore, the results of the electrical conductivity variation with temperature showed that the composites with different dispersions and covalent functionalizations of CNTs exhibited varying trends in current variation with temperature. Such findings might have critical implications in completing the electrical conduction mechanism of polymer composites.

## Figures and Tables

**Table 1:** The fitted parameters of the field emission equation for the LDPE/CNT composites prepared by different methods.

Specimens	*A*	*B*	*n*	Adj. R-Square
CNT-120-4.5	0.00141	113.86	1.05	0.9964
CNT-Solution-4.5	0.00161	31.68	1.08	0.9977
CNT-60-6	0.00158	40.33	1.06	0.9934
CNT-120-6	0.010	18.79	1.09	0.9942
CNT-Solution-6	0.122	0	—	0.9946

## Data Availability

The raw/processed data required to reproduce these findings cannot be shared at this time as the data also forms part of an ongoing study.
